# Challenges in bridging the gap between protein structure prediction and functional interpretation

**DOI:** 10.1002/prot.26614

**Published:** 2023-10-18

**Authors:** Mihaly Varadi, Maxim Tsenkov, Sameer Velankar

**Affiliations:** ^1^ Protein Data Bank in Europe, European Molecular Biology Laboratory, European Bioinformatics Institute (EMBL‐EBI), Wellcome Genome Campus Hinxton Cambridge UK

**Keywords:** AI‐based structure prediction, AlphaFold, functional interpretation, PDB, protein structures, structural bioinformatics

## Abstract

The rapid evolution of protein structure prediction tools has significantly broadened access to protein structural data. Although predicted structure models have the potential to accelerate and impact fundamental and translational research significantly, it is essential to note that they are not validated and cannot be considered the ground truth. Thus, challenges persist, particularly in capturing protein dynamics, predicting multi‐chain structures, interpreting protein function, and assessing model quality. Interdisciplinary collaborations are crucial to overcoming these obstacles. Databases like the AlphaFold Protein Structure Database, the ESM Metagenomic Atlas, and initiatives like the 3D‐Beacons Network provide FAIR access to these data, enabling their interpretation and application across a broader scientific community. Whilst substantial advancements have been made in protein structure prediction, further progress is required to address the remaining challenges. Developing training materials, nurturing collaborations, and ensuring open data sharing will be paramount in this pursuit. The continued evolution of these tools and methodologies will deepen our understanding of protein function and accelerate disease pathogenesis and drug development discoveries.

**Abbreviations**
CAD: contact area difference
CAID: critical assessment of intrinsic disorder
EM: electron microscopy
FAIR: findable, accessible, interoperable, reusable
GDT: global distance test
IDR: intrinsically disordered region
MSA: multiple sequence alignments
NMR: nuclear magnetic resonance
PAE: predicted aligned error
PDB: protein data bank
pLDDT: predicted local distance difference test
PPI: protein–protein interaction

## 1 INTRODUCTION

The advent of AI‐based protein structure prediction tools, such as AlphaFold 2,^1^ RoseTTAFold2,^2^ ESMFold,^3^ OmegaFold,^4^ OpenFold,^5^ and UniFold,^6^ has brought forth a transformative era in life sciences.^7^, ^8^ With their unprecedented ability to predict protein structures with remarkable accuracy, these tools have provided a great tool to accelerate research in numerous fields, from drug discovery^9^ and structure determination^10^, ^11^, ^12^ to bioinformatics^13^, ^14^, ^15^, ^16^, ^17^ and synthetic biology.^18^, ^19^ Undoubtedly, we have stepped into an era where the impact of structural biology has massively broadened, reaching more researchers and domains than ever before.^20^, ^21^

Although the successes are evident from the notable surge in the adoption of predicted structure models across scientific disciplines, it is easy to overlook their limitations. Experts in structural biology laid out the principles for correctly working with predicted models, the shortcomings and limitations in their application, and how they can be misused.^8^, ^22^, ^23^, ^24^ Terwilliger et al.^12^ demonstrate how to streamline and expedite the protein structure determination process with predicted models and use them as valuable hypotheses for expanding research questions. Similarly, Lowe^24^ expressed that knowledge of a protein structure is rarely a significant obstacle in advancing drug discovery efforts. Instead, it forms the basis for generating insights and guiding the design of novel compounds. Predicted structure models are not meant to serve as replacements for understanding protein function, as several limitations still need to be addressed, even in this new era of structural biology.

While we revel in this new age of abundant structural data, we must acknowledge and grapple with its challenges. Amongst them is the inherent limitation of next‐generation prediction tools in accurately predicting multi‐chain assemblies. Many proteins exist as multimers in their functional state or interact dynamically with other protein molecules, complicating the prediction of their three‐dimensional structure and subsequent functional interpretation. Predicted structure models also lack several components typically associated with a protein and essential for its function or fold. For example, they do not encompass the presence of various ligands they are often associated with, such as DNA, RNA, lipids, ions, and cofactors, thereby limiting their representation in their native state.^25^ There is also the absence of co‐ and post‐translational modifications, such as protein glycosylation, phosphorylation, acetylation, and several other covalent modifications.^26^

Additionally, a significant fraction of proteins and protein regions are intrinsically flexible, undergoing conformational changes as a part of their function.^27^ Most current prediction tools cannot capture this dynamic nature of proteins, often leading to static representations that might not accurately depict their biological reality. Furthermore, modern structure predictors cannot accurately predict mutations' structural effects.^22^ This limitation potentially restricts their applicability in areas like disease modeling, where understanding the structural implications of mutations is crucial.

Albeit the extensive challenges and limitations mentioned herein, there is still plenty of room for cautious optimism. It falls on the research community to infer functions from predicted structures.^28^ Indeed, protein structures, while serving as powerful tools in our scientific toolbox, are fundamentally coordinates in space. Yet, these structures can become more powerful tools for answering scientific questions with the necessary biological context from domain annotations and molecular context from ligands, cofactors, and ions.^29^ While our ability to fully understand and interpret the function of proteins, even those with accurately predicted structures continuously improves, one of the fundamental limitations of current AI‐based tools in structural biology is their inability to provide a comprehensive functional understanding based merely on a structure. Thus, while the predicted structures can help us better grasp protein function within certain limits, a protein's form alone is insufficient. We require additional biological and molecular context layers to tease apart the complex web of protein function.

As we venture further into this exciting era of structural biology, it becomes increasingly crucial to address this functional inference challenge. As a scientific community, we must develop strategies and scalable tools to help us bridge this gap between structure and function. By doing so, we can fully harness the potential of the vast trove of predicted structures, allowing us to gain deeper insights into the intricate workings of life at the molecular level.

One of the most groundbreaking aspects of these advancements is the democratization of protein structure data. AlphaFold, ESMFold and other tools are not only available as software but have also been used to launch new, dedicated databases, such as the AlphaFold Protein Structure Database^30^ and the ESM Metagenomic Atlas,^3^ which offer open access to a staggering wealth of more than 900 million predicted protein structures, immensely expanding our collective structural biology resource, potentially catalyzing significant strides in our understanding of life at the molecular level.

Another important consideration is that as predicted protein structures reach a wider audience, many researchers new to the field may need assistance with correctly interpreting protein structure data. This consideration emphasizes the need for comprehensive training materials and resources to prevent potential misinterpretations of the models. Establishing these data resources has been a significant effort, and for these to remain relevant and useful to the broader scientific community, they must continue to evolve to meet the needs across diverse research domains.

Finally, from a research data management point of view, another challenge lies in the necessity for a standardized way to access models from different data providers. Given the dominance of resources like AlphaFold DB and ESM Metagenomic Atlas, there is a risk that more minor predictors, which may excel in specific niches, could be overlooked. For example, a suite of tools predicting structures of proteins that make up various components of the adaptive immune system^31^ or a curated set of predicted protein structures from the ABC transmembrane protein family^32^ are valuable datasets which might be harder to find. A uniform system for accessing and comparing models across platforms could help alleviate this issue, bringing us to the role of initiatives such as the 3D‐Beacons Network, which seeks to provide a centralized platform for accessing protein structure models from various resources.^33^ Moreover, the common understanding between these data resource providers on making quality assessment metrics accessible is another consideration that makes this network valuable.

In the unfolding landscape of protein structure prediction, several critical challenges persist that need addressing. This perspective will delve into these hurdles, illuminating potential solutions and directions for further development in the field. Here, we aim to address these challenges, facilitating dialogue and fostering collaborative efforts to navigate this exciting new era of protein structure prediction.

### 1.1 Predicting multi‐chain structures: Current challenges

Despite the recent advances in single‐chain protein structure prediction, the challenge of accurately predicting multi‐chain structures remains a significant hurdle in structural biology.^34^, ^35^ Understanding the function of proteins that operate through transient or stable macromolecular interactions necessitates access to quaternary structures.^36^, ^37^, ^38^, ^39^ However, the number of experimentally resolved complexes is underrepresented since it is estimated that only 5% of human PPIs are structurally characterized.^40^

To tackle this challenge, the research community repurposed tools like AlphaFold2, to predict the structure of multimeric protein complexes, even though it was not initially designed to model such assemblies.^41^, ^42^ AlphaFold‐Multimer,^43^ designed to predict macromolecular complexes, operates at higher accuracy but still lags behind the accuracy of single‐chain models. It was also reported that the accuracy of predicted multimeric complexes declines with an increasing number of constituent structures.^44^ This degradation in performance arises from the escalating challenge of discerning coevolution with the addition of more protein chains as it increases the possible pairings of sequences from individual multiple sequence alignments (MSAs).^42^, ^44^, ^45^

**FIGURE 1:**
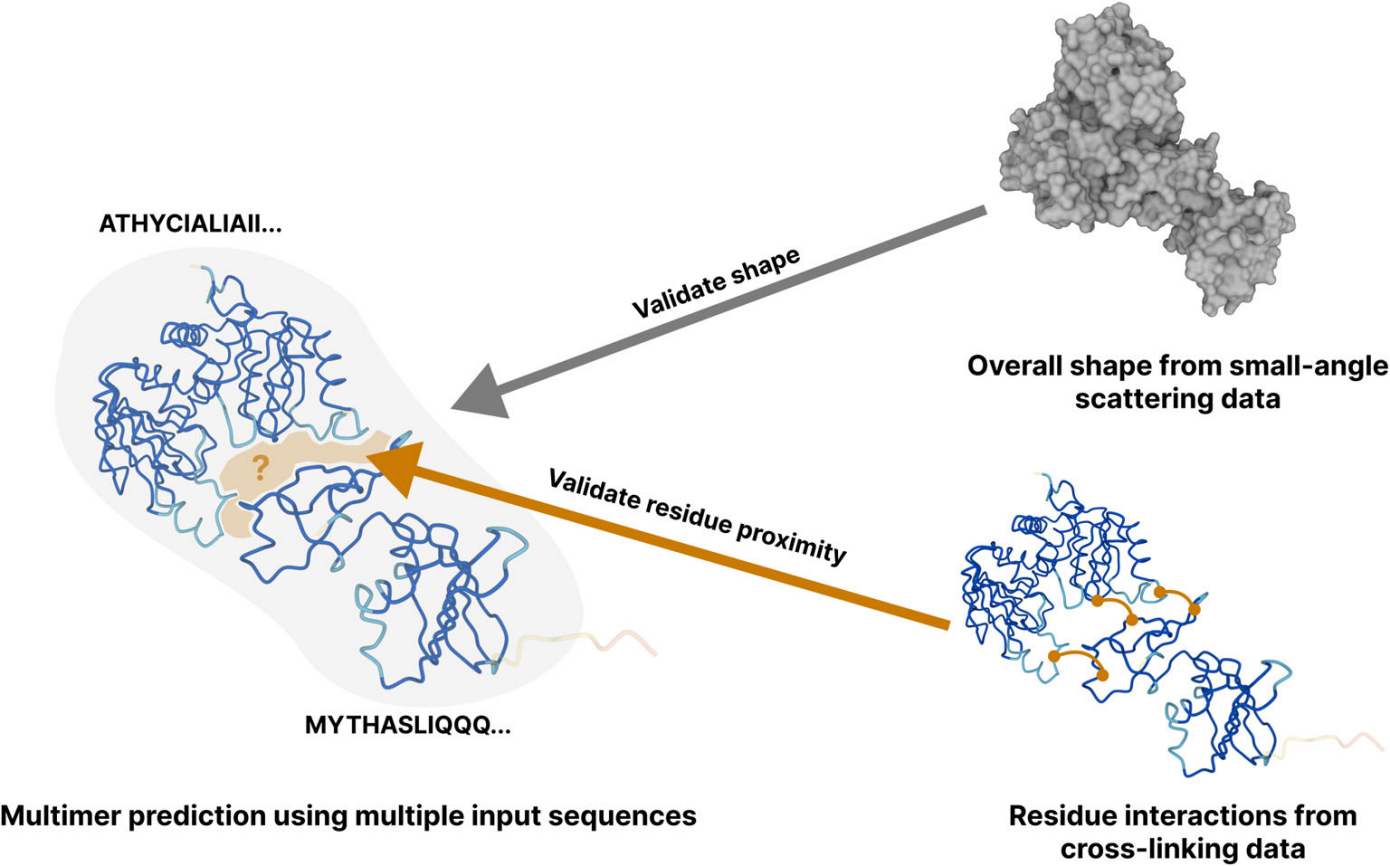
Validation of multimeric predictions using low‐resolution experimental data. This conceptual figure shows that external validation of computationally predicted models is generally preferable, but it becomes crucial when predicting the structure of multimeric assemblies. Experimental data such as small‐angle scattering and cross‐linking provide valuable information on the overall shape and the proximity between specific residues.

It is crucial to emphasize that given the lower accuracy of multi‐chain models, integrating additional experimental data becomes essential for validating these models (Figure 1), which underscores the usefulness of the predicted models in generating hypotheses and the indispensable role of experimental data in validating the hypothesis to enhance our understanding of protein function. By using the combination of experimental data and predicted models, the research community has predicted pairs of proteins on a small scale to structurally visualize protein–protein interactions (PPIs) supported by experimental data.^40^, ^44^ Other research groups have used predicted models as subcomponents to resolve behemoth assemblies, like the nuclear protein complex,^46^ guided by electron microscopy (EM) data.

A series of studies harness the sensitivity and depth of information from proteomics data; crosslinking and co‐fractionation mass spectrometry overcome the formidable computational scales of structurally characterizing PPIs.^35^ Cross‐linking data has proven invaluable for validating predicted assemblies.^47^ Tüting et al. (2023) similarly centered on uncovering protein communities and exploring inter‐protein interactions by using mass spectrometry‐derived proteomics data to identify protein assemblies and methodically fit AlphaFold models.^17^ The authors have the added advantage over multi‐chain predictors in that they can unambiguously capture near‐native states of protein complexes. Others in this research community have used the same methods in reverse, using databases with experimentally‐supported interactions to identify pairs of interacting proteins, structurally elucidate the interactome, and validate the predictions with crosslinking data.^40^ The approaches outlined offer an innovative solution to some of the limitations faced by multi‐chain protein structure tools like AlphaFold‐Multimer.^17^, ^47^ By utilizing proteomics data for identifying and validating protein communities, they can circumvent some ambiguities that often arise when predicting protein complex structures without prior knowledge.

Similarly, nuclear magnetic resonance (NMR) data has been utilized to corroborate multi‐chain model predictions. Notably, intermolecular contacts identified by Nuclear Overhauser Effect spectroscopy were combined with AlphaFold2‐Multimer predictions to generate an AZUL:UBA model structure.^48^

New developments in bioinformatics approaches have the potential to improve the accuracy of multi‐chain model predictions. Earlier methods for pairing interacting partners in MSAs were not yet optimal, and new methods might more reliably determine which potential interacting partners are orthologues that maintain the targeted interaction, compared to paralogues that do not.^49^

These advancements in multi‐chain prediction hold substantial practical implications for large‐scale interactome predictions.^50^ The AlphaPulldown package, a Python tool for PPI screens using AlphaFold‐Multimer,^51^ exemplifies this potential. Given the availability of accurate‐enough multi‐chain models, it offers a means for researchers to identify which proteins may interact based on their respective confidence metrics. In another proof‐of‐concept, Banhos Danneskiold‐Sams E et al. devised a computational approach to predict high‐confidence cell‐surface receptors for various ligands through structural binding prediction, which could greatly expand our understanding of cell–cell communication and holds broad applicability in biology.^52^

While the prediction of multi‐chain protein structures poses significant challenges, recent advancements and ongoing research offer hope for substantial progress. By integrating experimental data and refining prediction models, we inch closer toward a better understanding of macromolecular interactions.

### 1.2 Protein conformational states and flexibility: Unmet needs in structural predictions

**FIGURE 2:**
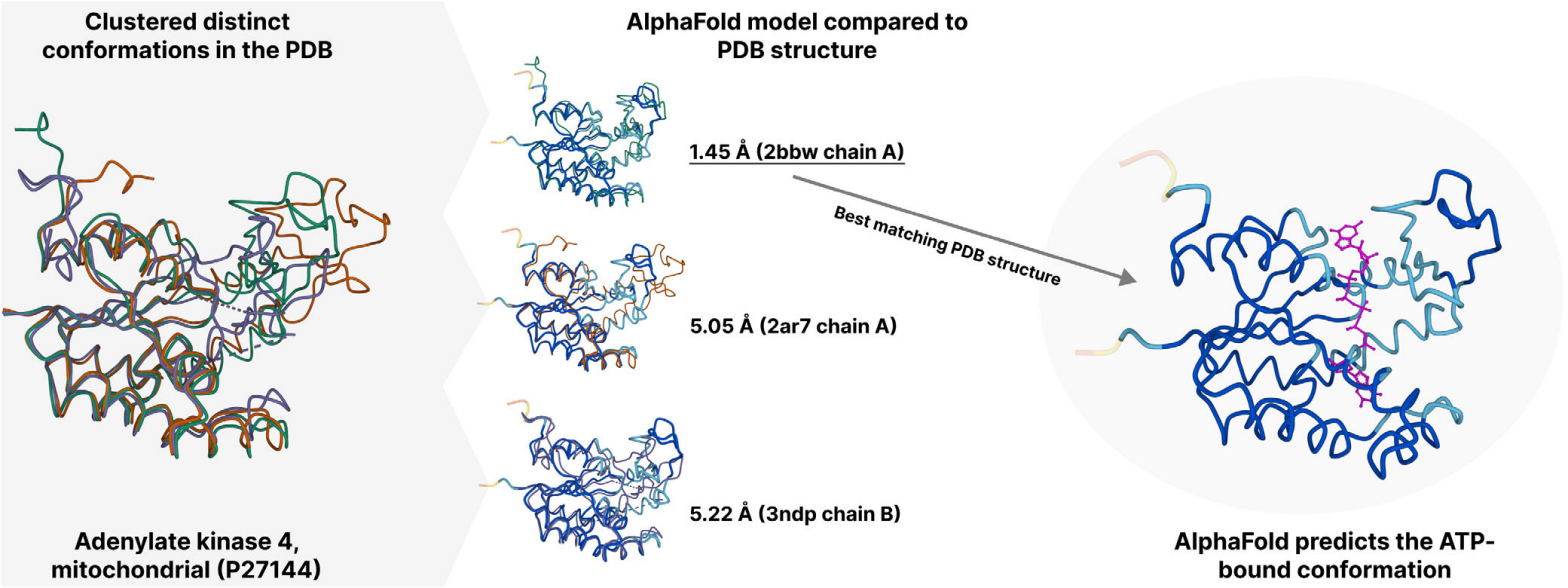
Comparing AlphaFold models to distinct conformations in the PDB.

AI‐based protein structure prediction tools typically generate static models that reflect a stable conformation of a given protein sequence. Proteins often have more than one distinct conformation, and the dynamic nature of proteins, particularly the alternation between active and inactive states, is crucial for their biological function.^26^ Calpain, a calcium‐dependent protease and a potential therapeutic target in cancer, oscillates between distinct conformations depending on their environment or binding status.^53^ Other proteins, like hexokinase, adopt different conformations based on their interaction with sugar molecules. In such cases, AlphaFold tends to predict a single conformation. Discerning whether this model represents an active or inactive state is only possible with additional context (Figure 2). In some databases, like PDBe‐KB,^29^ it is possible to superpose the AlphaFold models to distinct conformations observed in the experimentally determined structures available in the PDB for a protein of interest.

AlphaFold and most similar new‐generation protein structure prediction tools consistently predict a single conformation. It might be unclear which conformation it is if a protein has more than one distinct, biologically relevant conformation. In the example above, the adenylate kinase‐4 protein adopts significantly different conformations depending on its interaction with ATP. Based on data from the PDB, AlphaFold predicts the ATP‐bound conformation.

Inherently, AlphaFold 2 provides only a single conformation per protein sequence, but according to studies, there might be approaches to alleviate this limitation. A recent study by Stein and Mchaourab^54^ presents a novel method using in silico mutagenesis and MSAs to direct AlphaFold 2, facilitating it to yield alternate protein conformations. This approach might imply that by manipulating the input, the model might generate multiple meaningful structures, hinting at its adaptability and potential to represent the inherent flexibility of proteins, although more work is required to validate if the predicted conformations are biologically relevant. In a different approach, using the latest network of RosettaFold2,^2^ the work by Stein and Mchaourab^54^ raises the idea of combining the right components from different deep learning network architectures. The next generation of these computational methods may switch between predicting highly accurate protein structures with multiple conformations from evolutionarily informative MSAs or responsibly scaling to predict single conformations from large metagenomic datasets.^3^, ^55^ Such a modular approach could benefit from new developments such as the Distributional Graphormer, a deep learning‐based tool designed to predict the equilibrium distribution of molecular systems, offering a more efficient alternative to traditional, computationally intensive methods like molecular dynamics simulation.^56^

An intriguing observation is that the AI‐based new prediction methodologies often predict the conditional fold of proteins that exhibit intrinsically disordered regions (IDRs) in their free state. IDRs can adopt temporary secondary structures upon interacting with their macromolecular partners or under various physiological conditions. Notably, these folded regions often have high confidence scores, with pLDDT values exceeding 70, suggesting a more complex relationship between intrinsic disorder and lower pLDDT scores than previously understood.^57^ A recent study has systematically characterized human IDRs concerning pLDDT and its correlation to conditional folding, where 15% are predicted with high confidence.^58^ Based on their findings, AlphaFold could accurately identify 88% of intrinsically disordered proteins known to fold upon binding. Their work highlights impressive precision, considering this protein class is underrepresented in the AlphaFold training dataset. Indeed, as demonstrated by the latest critical assessment of intrinsic disorder predictors, although AlphaFold is not the best predictor of intrinsic disorder propensities, its prediction accuracy is already high. The main distinction among state‐of‐the‐art predictors lies in their runtime rather than accuracy.^59^ Despite these advancements, accurately modeling intrinsically disordered protein ensembles remains a formidable challenge. The achievement of this goal would significantly impact drug discovery, specifically in the targeting of IDPs with drug molecules based on structure models.^60^ This unmet need underscores the continued necessity for development and refinement in structural prediction in the context of conformational flexibility.

### 1.3 Assessing the quality and reliability of predicted structures

Quality assessment of predicted protein structures is essential to their practical application across a broad range of biological and biomedical research. It is crucial to understand the accuracy of the predictions and the areas of a model where confidence is high or low.^61^, ^62^, ^63^, ^64^, ^65^, ^66^, ^67^, ^68^ Several metrics, notably the global distance test,^69^ template modeling score,^70^ and superposition‐independent scores like local distance difference test,^71^ residue–residue contact area difference (CAD),^72^ and SphereGrinder^73^ have been developed to evaluate the quality of these structures by comparing them to experimentally‐resolved structures, providing valuable indications of their reliability.

AlphaFold and other predictive tools generate predicted quality metrics as output, aiding users in interpreting the predicted protein structures. AlphaFold, for instance, provides two metrics: per‐residue confidence scores (pLDDT) and Predicted Aligned Error (PAE).^1^ The pLDDT score provides an estimate of the confidence of the network in the individual residue in the predicted model, with scores below 50 often indicating regions with low confidence and those above 90 suggesting a high degree of confidence.^74^ On the other hand, PAE estimates the predicted error estimate in the relative position of residue pairs.

However, while these metrics offer valuable tools in the interpretation of the predicted model, they have limitations. They may not accurately predict the level of uncertainty in regions of intrinsic disorder or flexible loop regions, underscoring the need for additional lines of evidence to validate these models.^7^ In a large‐scale analysis, Ruff and Pappu^75^ outlined that regions with a low pLDDT score average do not always necessarily indicate a failure of AlphaFold, but rather a reflection of the conformational heterogeneity. Later analysis found that this holds for specific categories of intrinsic disorder, such as flexible linkers and entropic chains, but IDRs that adopt a stable secondary structure when bound to an interaction partner are generally predicted with higher (>70) pLDDT scores.^57^ In another analysis, Monzon et al.^76^ used AlphaFold to predict the structures of short sequences comprising AntiFam, which stores Pfam families for protein sequences derived from spurious open reading frames. The authors initially attempted to confirm these protein sequences did not fold into familiar globular proteins for their quality control purposes. Instead, they discovered a negative correlation between sequence length and average pLDDT score, where shorter sequences displayed higher average pLDDT scores, which might indicate that this potential bias is important to consider when using predicted structures for short sequences. The AntiFam resource may serve as a valuable negative control dataset to assess the accuracy of pLDDT, including metrics from other deep learning methods.

Experimental data are crucial in validating predicted structures.^7^ Predictions can be compared with experimental structures determined by X‐ray crystallography, NMR spectroscopy, cryo‐EM, as well as low resolution and specialist techniques such as cross‐linking data and small‐angle scattering information to gauge their accuracy at different levels (^35^, ^77^; Q.^78^). These experimental techniques can validate the predictions and offer insights into protein dynamics, providing a level of detail that predictions alone often cannot capture. Integrating multiple lines of evidence when assessing the quality and reliability of predicted structures is thus of utmost importance, with validation by comparing computational predictions to experimental data being the preferred option.

Indeed, while many methodologies are emerging to gauge the quality of predicted protein structures, more must be done in developing tools that evaluate the robustness of these prediction methods.^79^, ^80^ Techniques that employ adversarial strategies,^81^, ^82^ commonly utilized in fields like computer vision and natural language processing,^83^, ^84^ are conspicuously absent in our current arsenal of evaluation techniques.

Revisiting the effective integration of structural proteomics and Alphafold‐Multimer, a significant advancement is integrating structural proteomics data with PAE scores, as visualized in a recently released PAE viewer.^85^ Such innovations pave the way for enriching the functional capabilities of visualizing and interacting with predicted protein complexes. Crucially, the introduction of associated crosslinking data exemplifies how to evaluate the reliability of complexes using the PAE plot and, thus, potentially offers a standard for others to adopt.

There is a pressing need for the continued development and standardization of tools and metrics to assess predicted protein structures' quality and reliability and the underlying methods that generate them.^67^, ^86^ An emphasis should be on adapting current methods and developing new ones for estimating the model accuracy of multi‐subunit protein complexes.^2^, ^3^, ^4^, ^5^, ^6^ Ongoing efforts in this direction will foster trust in computational predictions and provide the necessary metric for their careful usage across diverse fields, thus extending our understanding of protein structure and function.

### 1.4 Understanding the consequences of mutations on protein stability and function

The study of how mutations influence protein stability and function carries critical implications across diverse scientific domains, including disease research, drug discovery, and protein engineering.^7^, ^8^, ^19^ Mutations can profoundly affect a protein's properties^87^, ^88^ and its structure, instigating significant conformational changes and even the manifestation of diseases.^89^, ^90^

Alterations in protein stability resulting from mutations can disrupt the intricate network of internal contacts, leading to changes in protein folding. These modifications may result in the protein's misfolding, often associated with deleterious outcomes such as neurodegenerative disorders.^91^ The functional implications of mutations are just as pivotal. By altering active sites or modifying ligand binding sites, allosteric sites and mutations can significantly affect a protein's interaction with other molecules, causing detrimental functional changes. For instance, a mutation in the active site of an enzyme can disrupt its catalytic activity, impacting the metabolic pathway in which the enzyme is involved.^92^, ^93^

Computational models generally do not directly support the prediction of the impacts of mutations on protein structures.^1^, ^22^ However, the Baker group has recently showcased a refined method for mutation impact prediction.^94^ They leveraged RoseTTAFold Joint, integrating sequence and structural data, which amplified the model's capacity to help researchers understand the protein mutational landscapes and potentially enhance precision in mutation effect predictions. However, such a perspective alone could be restrictive since single‐point mutations' impact is only sometimes evident from studying the corresponding single protein in isolation.^40^, ^87^, ^95^ However, other tools like FoldX and Missense3D can use predicted protein structures as input and may produce improved predictions in the context of structural stability changes as a consequence of mutations.^96^, ^97^

An example of a successful application of predicted protein models in characterizing pathogenic mutations with no experimentally‐determined structures or close homologs was demonstrated recently.^95^ Among the hundreds of proteins studied, 80% of pathogenic mutations were found close to predicted functional sites, such as ligand‐binding and conserved sites and protein–protein interfaces in predicted structure models. Altogether, by placing missense mutations within a structural context, their pathogenic nature could more effectively be deciphered. This approach underscores the crucial role of structures in understanding missense mutations and highlights the importance of predicted structure models, in the absence of experimentally‐resolved structures.^98^ Another example of this approach is AlphScore,^99^ a cutting‐edge pathogenicity prediction score derived from AlphaFold. This tool is calibrated on key feature classes, including pLDDT, physicochemical descriptors, amino acid networks, and solvent accessibility. When used with prevalent mutation predictors, AlphScore delivers an enriched, multi‐dimensional perspective of mutation impacts, enhancing our understanding of their consequences on protein function and stability. These approaches highlight the importance of having a rich ecosystem of complementary computational tools to address the limitations of individual methods to help tackle challenging biological problems.

### 1.5 The role of interdisciplinary collaborations and training in advancing structural biology

In an era where computational and experimental biologists can collaboratively interrogate biological systems, using predicted structures has become an accessible and versatile tool across the life sciences for understanding protein function. Recent advances in accurate protein structure prediction have paved the way for such investigations, even in fields like metagenomics that previously did not significantly utilize structural data.^3^

As protein structures permeate a broader range of scientific investigations, supporting new users in navigating this rich resource becomes imperative. It falls on the shoulders of structure prediction tool providers and structure data resources to ensure that users understand the best practices of working with protein structures and know the limitations, which might be unique to specific prediction software or more generic.

**FIGURE 3:**
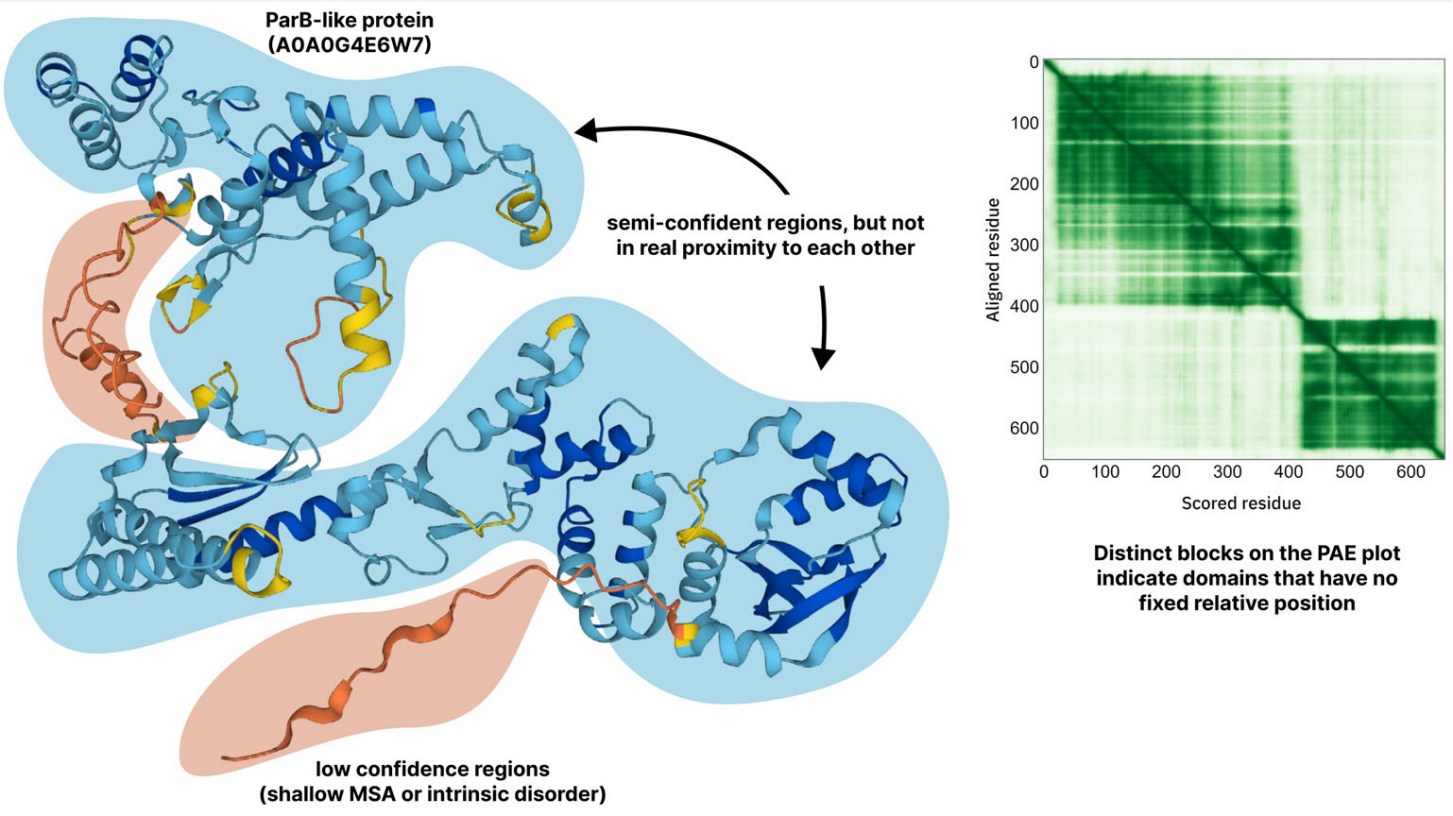
Most frequently used confidence metrics. The most frequently used confidence metrics of the new AI‐based protein structure prediction tools are the pLDDT and PAE scores. The pLDDT scores are local confidence metrics, while the PAE scores give information about the confidence in the relative position of residue pairs.

A crucial aspect of this education is the understanding and consideration of available confidence metrics associated with modeled structures (Figure 3). As discussed in a previous section, users of AlphaFold models should be well‐versed with metrics such as the pLDDT local confidence metric and the predicted aligned error (PAE) confidence metric. The pLDDT score can help identify regions of the predicted model that are predicted with low confidence, which in many cases represent IDRs but can also reflect limitations in the input data, for example, shallow multiple sequence alignment. The PAE provides insights into the network's confidence in the spatial relationships between residue pairs, which can help identify domains, offering snapshots of possible conformations and suggesting potential flexibility. Both metrics are essential in making informed interpretations and decisions based on predicted structures. Complexities arise in certain situations, such as membrane proteins, where flexible loops may occupy spatial positions that typically represent the membrane location. Understanding such intricacies is fundamental to accurately working with these data.

While some training materials are available from data providers and academic groups, there remains a need for a more centralized, systematic library of structural biology training materials that encompass computational and experimental models and their use.

### 1.6 The power of data sharing and integration in protein structure predictions

The impact of biological data, including predicted structures, relies significantly on the accessibility and integration of data. Ensuring findable, accessible, interoperable, and reusable (FAIR) access to both computationally‐predicted and experimentally determined protein structures from diverse providers enables the broader scientific community to tackle specific biological questions.

Currently, three primary databases, the ESM Metagenomic Atlas (600million+), the AlphaFold Protein Structure Database (200million+), and SWISS‐MODEL (>2 million)^100^ provide predicted structure models. However, more specialized databases exist that can provide more pertinent models for specific problems (Table 1). For example, AlphaFill^25^ offers the AlphaFold database models with obligatory ligand molecules. The ModelArchive^101^ contains invaluable datasets of predicted proteins, including complexes, while the Protein Ensemble Database^102^ offers conformational ensembles derived using experimental data with computational modeling, of intrinsically disordered proteins. The challenge users face is to keep track of these diverse data providers and discover the models that best meet their needs. Initiatives such as the 3D‐Beacons Network significantly democratize access to protein structures by providing FAIR, standardized data access mechanisms for macromolecular structure files from various data providers while also striving to provide standard validation metrics.^33^

**TABLE 1:** Data resources of predicted protein structures.

Data resources	Description
3D‐Beacons Network https://3d‐beacons.org	Unified access to 220 million + experimentally derived and predicted protein structures and their quality metrics
AlphaFold Protein Structure Database https://alphafold.ebi.ac.uk	214 million + predicted monomeric protein structures
AlphaFill https://alphafill.eu/	1 million AlphaFold models with transplanted small molecules
CHESS Human Protein Structure Database https://www.isoform.io/	230k + predicted human protein structures
ESM Metagenomic Atlas https://esmatlas.com/	600 million + predicted monomeric protein structures
ModelArchive https://www.modelarchive.org/	74k + predicted structures
Protein Ensemble Database https://proteinensemble.org/	300k + ensemble conformations
Protein Data Bank in Europe—Knowledge Base https://pdbe‐kb.org	80 million + structure‐based annotations
SWISS‐MODEL Repository https://swissmodel.expasy.org/repository	2.3 million + predicted protein structures

From a broader perspective, it is vital to enriching these structures, whether predicted or experimentally determined, with biological context to enhance their interpretation and utilization. This goal can be achieved by annotating structures with structural, functional, and biophysical attributes, as the PDBe‐KB database aims to facilitate, or by providing services that simplify the transfer of these annotations between models. Several tools assist users with searching and superposing structures from databases such as the Protein Data Bank and the AlphaFold database. Structural clustering of all structure models in the AlphaFold database has been demonstrated as a powerful resource for studying protein function and evolution across the Tree of Life.^15^ Foldseek (van Kempen et al., 2023) and DALI offer structure‐based search functionality and, together with other initiatives, help illuminate “functional darkness” in the UniProt and AlphaFold databases, allowing for the exploration of novel protein families and structural folds.^14^

## 2 CONCLUSION

AI‐based protein structure prediction software, notably highlighted by tools like AlphaFold 2, RoseTTAFold, ESMFold, and many others, have ushered in a new era in (structural) biology. These tools can identify previously unknown protein families and folds, thereby broadening our understanding of the evolutionary landscape of proteins.^103^

Several studies exemplify how predicted structures can effectively address real biological challenges. For example, in a recent study, Huang et al. generated predicted structure models for the entire deaminase family of proteins using AlphaFold 2 and performed structure‐based clustering.^16^ This approach identified new members of existing clades and revealed that many proteins within this family were not traditional double‐stranded DNA cytidine deaminases. Impressively, they were able to engineer one of the minor proteins of the new clade members, enabling successful cytosine base editing in soybean plants, demonstrating the potential of these methods to drive advances in practical applications such as agriculture.

We must, however, acknowledge the challenges that lie ahead. While we now have access to an unprecedented amount of structural data, interpreting the function of proteins based solely on their form is not trivial. There is a critical need for additional context from experimental data, domain annotations, molecular interactions, and the dynamics of protein flexibility. Nonetheless, the structural biology and bioinformatics community, with decades of experience in working with protein structures, is well‐positioned to meet these challenges. The responsibility of structure data providers is to aid researchers across the life sciences to use this rich dataset to accelerate fundamental and translational research.

The advancements in AI‐driven structural prediction mark the onset of a golden age in structural biology. As we navigate this exciting landscape, we remain optimistic about the potential of these tools to revolutionize our understanding of protein structure and function and to advance our ability to address complex biological problems. The future of structural biology is undoubtedly bright, and we look forward to the discoveries it holds.

## AUTHOR CONTRIBUTIONS

**Mihaly Varadi:** Conceptualization; investigation; writing – review and editing; writing – original draft; project administration; supervision. **Maxim Tsenkov:** Writing – original draft; investigation; writing – review and editing; validation. **Sameer Velankar:** Funding acquisition; conceptualization.

## CONFLICT OF INTEREST STATEMENT

The authors declare no conflict of interest.

### PEER REVIEW

The peer review history for this article is available at https://www.webofscience.com/api/gateway/wos/peer-review/10.1002/prot.26614.

## Data Availability

Data sharing is not applicable to this article as no new data were created or analyzed in this study.
